# Features of a novel protein, rusticalin, from the ascidian *Styela rustica* reveal ancestral horizontal gene transfer event

**DOI:** 10.1186/s13100-019-0146-7

**Published:** 2019-01-19

**Authors:** Maria A. Daugavet, Sergey Shabelnikov, Alexander Shumeev, Tatiana Shaposhnikova, Leonid S. Adonin, Olga Podgornaya

**Affiliations:** 1 Laboratory of Cell Morphology, Institute of Cytology, Russian Academy of Sciences, St. Petersburg, Russia; 2 Department of Intracellular Signaling and Transport, Institute of Cytology, Russian Academy of Sciences, St. Petersburg, Russia; 3 Department of Cytology and Histology, St. Petersburg State University, St. Petersburg, Russia; 4 School of Biomedicine, Far Eastern Federal University, Vladivostok, Russia; 5 Laboratory of Evolutionary Morphology, Zoological Institute, Russian Academy of Sciences, St. Petersburg, Russia

**Keywords:** Ascidians, Bacteriophage, Hemocytes, Horizontal gene transfer, L-alanyl-D-glutamate peptidase, Trichoplax, tRNA

## Abstract

**Background:**

The transfer of genetic material from non-parent organisms is called horizontal gene transfer (HGT). One of the most conclusive cases of HGT in metazoans was previously described for the cellulose synthase gene in ascidians.

**Results:**

In this study we identified a new protein, rusticalin, from the ascidian *Styela rustica* and presented evidence for its likely origin by HGT. Discernible homologues of rusticalin were found in placozoans, coral, and basal Chordates. Rusticalin was predicted to consist of two distinct regions, an N-terminal domain and a C-terminal domain. The N-terminal domain comprises two cysteine-rich repeats and shows remote similarity to the tick carboxypeptidase inhibitor. The C-terminal domain shares significant sequence similarity with bacterial MD peptidases and bacteriophage A500 L-alanyl-D-glutamate peptidase. A possible transfer of the C-terminal domain by bacteriophage was confirmed by an analysis of noncoding sequences of *C. intestinalis* rusticalin-like gene, which was found to contain a sequence similar to the bacteriophage A500 recombination site. Moreover, a sequence similar to the bacteriophage recombination site was found to be adjacent to the cellulose synthase catalytic subunit gene in the genome of *Streptomices* sp*.*, the donor of ascidian cellulose synthase.

**Conclusions:**

The C-terminal domain of rusticalin and rusticalin-like proteins is likely to be horizontally transferred by the bacteriophage A500. A common mechanism involving bacteriophage mediated gene transfer can be proposed for at least two HGT events in ascidians.

## Background

Ascidians are marine benthic animals from the subphylum Tunicata (Urochordata), which is considered the closest living sister group to vertebrates based on genome analysis [1]. The name *Tunicata* derives from the unique exoskeleton of these animals, the tunic, comprising both proteins and carbohydrates [2]. A remarkable feature of tunicates is biosynthesis and incorporation of cellulose into their tunic. The ascidian life cycle includes a mobile larva possessing a notochord and a sessile filter-feeding adult stage [3]. Ascidians harbor diverse microbiota [4], and their cellulose synthase is thought to have been acquired by horizontal gene transfer (HGT) from the bacterial *Streptomyces* sp*.* genome [5, 6]. The adaptive importance of HGT is supported by studies showing that mutants of cellulose synthase exhibit defects in metamorphosis and maintaining a sessile lifestyle, suggesting that it was an acquisition of cellulose synthesizing ability that permitted ascidians to evolve their sessile lifestyle [7].

Most of the described cases of HGT between prokaryotes and eukaryotes are thought to have involved transfer of genes from former to the latter [8, 9]. Possessors of former prokaryotic genes include multicellular animals [10] and, in particular, chordates [11, 12]. The fraction of horizontally acquired genes in a eukaryotic genome can reach 8%, as was described for the bdelloid rotifer *Adineta vaga* [13]. It has been shown that some of these horizontally transferred genes are expressed and produce functional protein products [14, 15]. Possible mechanisms of HGT between prokaryotes and eukaryotes are widely discussed, with viruses being considered as the most probable vectors of transmission into the genome [16, 17]. The existence of nuclear localization signals in bacteriophage proteins covalently bound to viral DNA lends support to this hypothesis. Facilitation of gene delivery into the eukaryotic nucleus by these signal sequences has been confirmed experimentally [18]. A broad range of gene engineering techniques adopting virus vectors for eukaryotic cells transformation in vitro and in vivo [19, 20] may provide further evidence in support of this hypothesis.

Compelling evidence supports the HGT of the cellulose synthase gene of the ascidian *Ciona intestinalis* [5]. This gene is expressed in the tunic-producing epidermis [7, 21]. Apart from epidermal cell layer tunic formation involves also blood cells [22, 23]. Several morphotypes of blood cells have been described for ascidians [22, 24–27], including hyalinocytes. In the blood of a solitary ascidian *Styela rustica* hyalinocytes and morula cells are two dominating cell groups, with an average abundance of 38 and 56%, respectively [22]. Hyalinocytes are characterized by the presence of numerous small granules. Their density is low, and so they can be separated by density gradient centrifugation [23, 28].

In this work we describe a novel protein, rusticalin, isolated from hyalinocytes of *S. rustica* and discuss its possible origin by HGT.

## Results

### cDNA cloning and sequence analysis

**Fig. 1:**
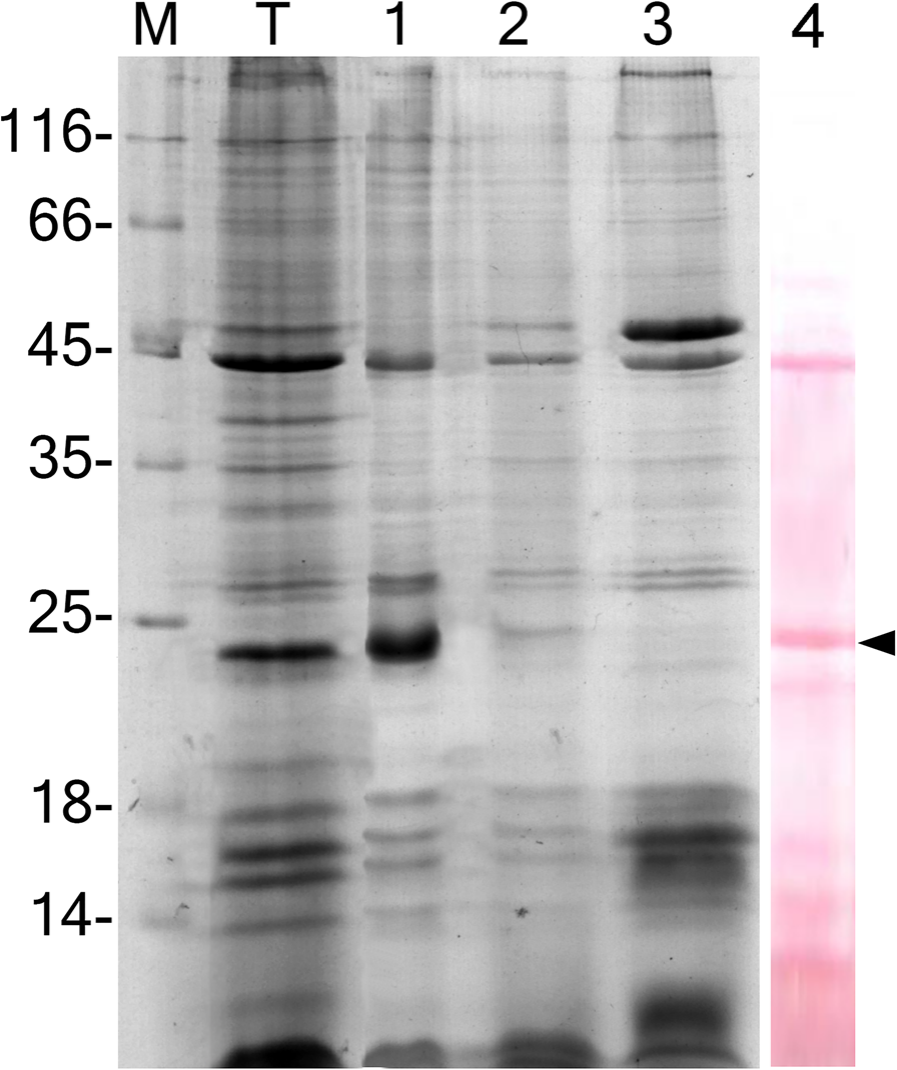
SDS-PAGE of blood cells proteins of ascidian *Styela rustica*. Lane T: whole blood cells; lane 1: the upper fraction of blood cells from discontinuous percoll gradient (above 35% percoll); lane 2: the intermediate fraction (between 35 and 45% percoll); lane 3: morula cells fraction from discontinuous percoll gradient (between 45 and 60% percoll); lane 4: whole blood cells proteins transferred to PVDF membrane and stained with Ponceau S. The major band of the upper (hyalinocytes) fraction of 23 kDa (black arrow) subjected to further matrix-assisted laser desorption/ionization tandem mass spectrometry MALDI MS/MS analysis. M – Molecular weight markers in kDa

**Fig. 2:**
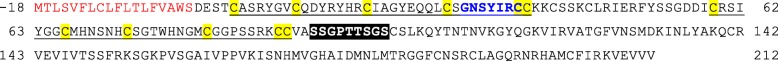
Deduced amino acid sequence of rusticalin. The predicted signal peptide is printed in red, the internal repeats detected with REPRO and RADAR are underlined, with cysteines shaded yellow, and a stretch of residues with relative solvent accessibility > 50% shaded black. Peptide sequence detected by de novo sequencing is printed in blue

Whole blood cells were separated by discontinuous percoll gradient and analyzed by SDS-PAGE (Fig. 1). The upper fraction above 35% percoll containing mainly hyalinocytes showed a major protein band of 23 kDa on SDS-PAGE. This band was subjected to trypsin digestion and MS/MS de novo sequencing, yielding a 7-residue-long peptide GNSYIRC. As a first attempt to find homologous proteins in databases the peptide was queried by tBLASTn against EST DataBase limited to Tunicata or without limitations, but showed a lack of reliable similarity. Therefore, the sequence information was used to design degenerate primers and to amplify full-length rusticalin cDNA through 3′ and 5′ Rapid Amplification of cDNA Ends (RACE) PCR. The rusticalin cDNA was 1002 bp, comprising a 5′-untraslated region of 111 bp, an open reading frame of 690 bp and a 3′-untraslated region of 201 bp. The first ATG at position 94–96 was assigned as the start codon. Two polyadenilation signals (AATAAA) were found 23 and 112 bp upstream of the poly(A) + tail. The ORF encoded a protein of 230 amino acid residues, including predicted signal peptide of 18 amino acid residues (Fig. 2, GenBank accession number MH115429).

The mature protein comprises 212 amino acid residues with a theoretical molecular mass of 23,309.7 Da. It contains 11 negatively charged residues (Asp + Glu) and 29 positively charged residues (Arg + Lys) yielding a calculated pI of 9.33. N-terminal region of rusticalin was found to contain two repeats, 34 and 33 residues in length, with a common cysteine spacing motif Cx_6_Cx_6-7_Cx_8_Cx_7_CC (Fig. 2). These protein regions are further referred to as cysteine-rich repeats.

**Fig. 3:**
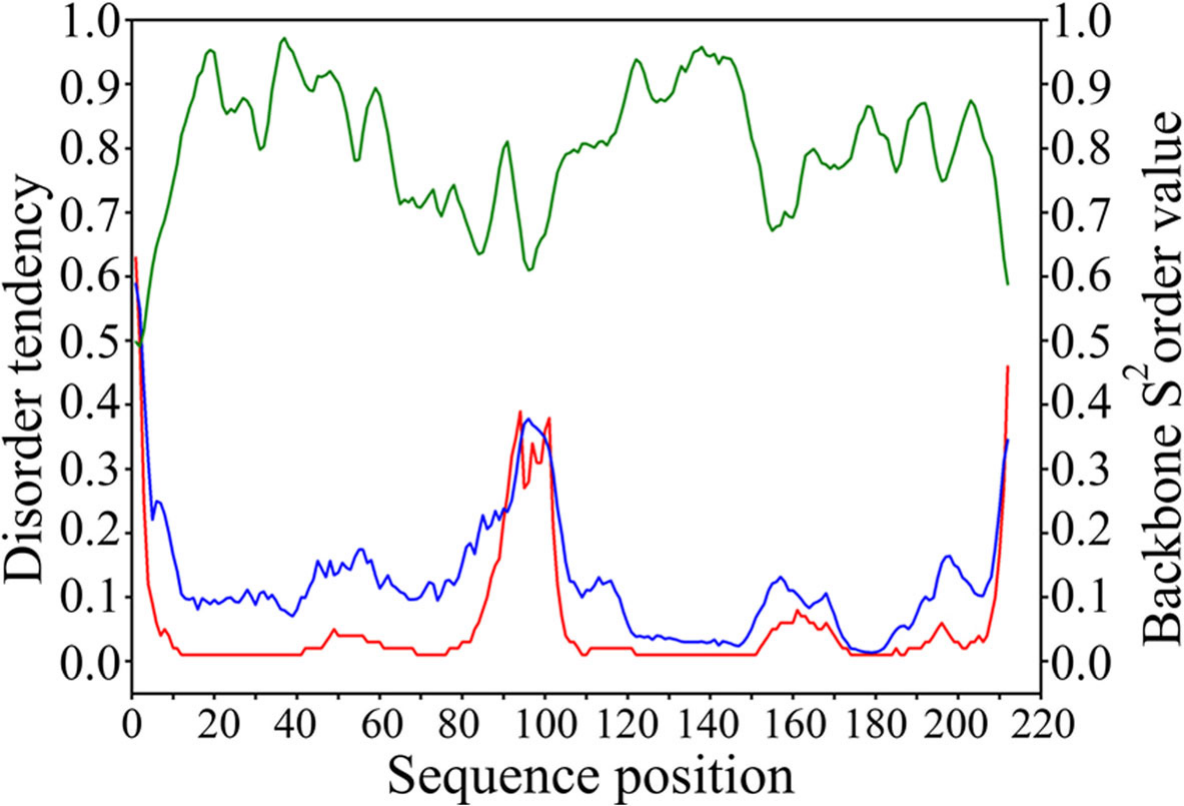
Prediction of disorder. Disordered regions predicted using Disopred3 (red line), SPOT (blue line) and protein backbone dynamics predicted using DynaMine (green line) for *Styela rustica* rusticalin

Computational tools were applied to detect putative domains and to predict relative solvent accessibility and disorder in the sequence. Scooby-domain tool predicted two domains with a boundary at Ser95. Prediction of the relative solvent accessibility revealed a stretch of eight highly exposed residues at positions Ser94-Ser102. Protein backbone disorder prediction showed the existence of two rigid regions linked by a short flexible region (Fig. 3). These data suggest that the hydrophilic and flexible region identified may be involved in the formation of a linker about six amino acid residues long connecting N- and C-terminal domains.

### Localization of rusticalin mRNA

**Fig. 4:**
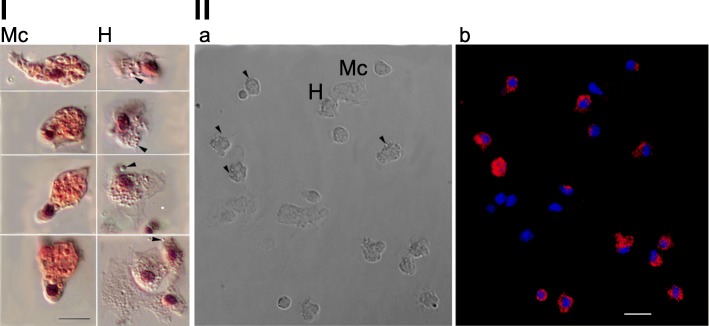
FISH detection of rusticalin mRNA in the blood cells. **I** Hyalinocytes (H) and morula cells (Mc), hematoxylin and eosin staining, DIC microscopy. Note hyalinocytes containing numerous small spherical granules (arrows). Scale bar, 5 μm. **II** Confocal sections showing the distribution of transcripts (b) within the cells with the morphology of hyalinocytes (a). Scale bar, 10 μm. The hybrids with Probe 2 were detected with streptavidin-Alexa594 (red pseudo color). 4′-6-Diamidino-2-phenylindole (DAPI) was used as a general DNA dye (blue pseudo color)

The localization of rusticalin mRNA in the blood cells was examined with fluorescent in situ hybridization (FISH). Confocal microscopy showed that flattened cells containing numerous small spherical granules were labeled (Fig. 4II, III). These cells were clearly identified as hyalinocytes based on the presence of characteristic granules (Fig. 4Ia), which were absent in morula cells (Fig. 4Ib). Thus the hybridization signal was restricted to the cytoplasmic space of hyalinocytes (Probe 2: Fig. 4IIb). The same results were observed with Probe 1; the negative control showed no hybridization signal (data not shown).

### Similarity search

**Fig. 5:**
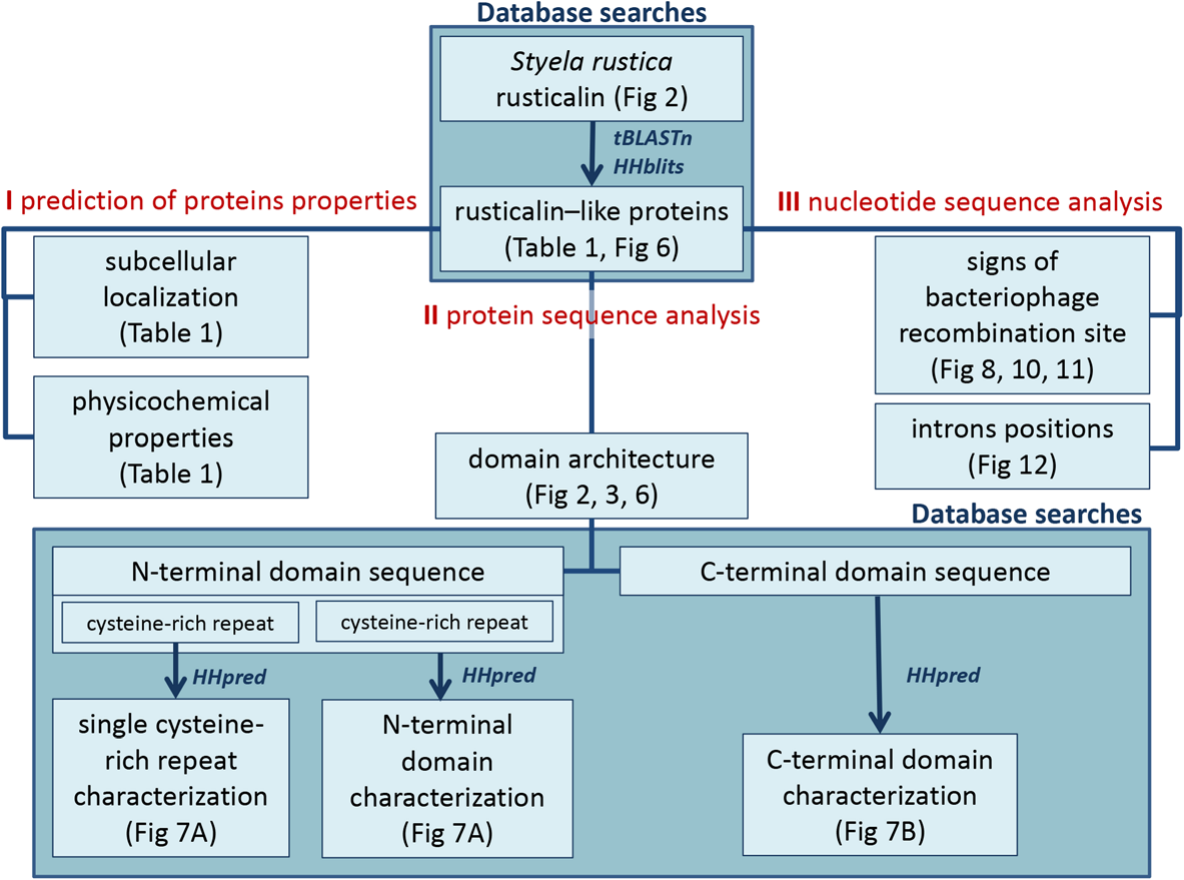
Workflow of rusticalin sequence analysis

The workflow of rusticalin sequence analysis is shown in Fig. 5. Searches against transcriptome and non-redundant protein databases using tBLASTn for finding close homologues and HHblits for remote similarity search identified discernible rusticalin-like proteins only in tunicates (*Oikopleura dioica*, *Ciona intestinalis*, *Ciona savignyi*, *Diplosoma listerianum*, and *Botryllus schlosseri*), cephalochordates (*Branchiostoma floridae* and *Asymmetron lucayanum*) and basal multicellular animals (coral *Alveopora japonica* and placozoan *Trichoplax adhaerens*) (Table 1). Multiple sequence alignment of all newly identified rusticalin-like proteins was queried against UniProtKB and the NCBI non-redundant protein databases. No other significant hits containing both predicted structural domains and covering more than 90% of a query were found. These results indicate that rusticalin-like proteins are taxonomically restricted to placozoans, corals, and basal chordates.

**Table 1:** Results of iterative search strategy used to identify rusticalin-like proteins

Species	Identification method	Sequence ID	Protein existence	Preprotein aa	Predicted cellular location	Predicted signal peptide	Putative mature protein		
							Molecular mass Da	pI	DE/RK
*Botryllus schlosseri*	TBLASTN	gb|JG375642.1| gb|JG376766.1|	transcript	271	extracellular	1–19	26,843	4.30	27/10
*Ciona intestinalis*	HHblits	XP_002128942.1	predicted ORF	271	extracellular	1–16	28,004	8.36	24/29
*Ciona intestinalis*	HHblits	XP_002122335.1	predicted ORF	267	extracellular	1–17	26,759	4.32	27/12
*Ciona intestinalis*	TBLASTN	GBKV01021559.1	transcript	271	extracellular	1–16	28,184	7.84	27/29
*Ciona savignyi*	TBLASTN	GGEI01017847.1	transcript	271	extracellular	1–15	27,988	8.06	26/29
*Diplosoma listerianum*	TBLASTN	gb|CN499160.1| gb|CN499008.1|	transcript	267	extracellular	1–17	26,889	4.39	28/12
*Oikopleura dioica*	TBLASTN	emb|FP744447.1|	transcript	263	extracellular	1–14	27,192	5.67	29/24
*Oikopleura dioica*	TBLASTN	GCJN01018270.1	transcript	260	extracellular	1–16	26,642	5,96	28/25
*Asymmetron lucayanum*	TBLASTN	GESY01031038.1	transcript	264	extracellular	1–16	26,489	5.43	26/20
*Branchiostoma floridae*	HHblits	XP_002588042.1 dbj|BW723234.1|	predicted ORF transcript	268	extracellular	1–15	27,117	5.10	28/20
*Branchiostoma floridae*	TBLASTN	GETA01020221.1	transcript	270	extracellular	1–16	27,235	5.01	30/21
*Alveopora japonica*	TBLASTN	GGJR01103857.1	transcript	269	extracellular	1–18	27,585	4.29	29/12
*Alveopora japonica*	TBLASTN	GGJR01116144.1	transcript	256	extracellular	1–18	25,764	4.23	29/12
*Trichoplax adhaerens*	HHblits	XP_002117795.1 gb|GR416119.1|	predicted ORF transcript	248	extracellular	1–18	24,575	7.51	13/14

**Fig. 6:**
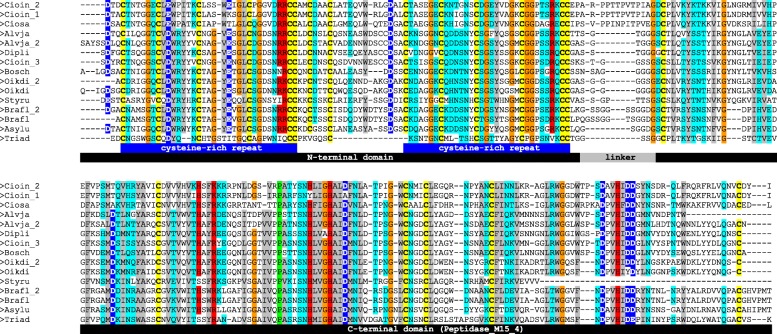
Multiple sequence alignment of rusticalin-like proteins. Predicted signal peptides were trimmed out before alignment. Note the perfect match and conservation of cysteine residues highlighted in yellow. N- and C-terminal domains of rusticalin-like proteins are shown in black. Note the lack of 40 C-terminal residues in rusticalin. The tentative linker region between N- and C-terminal domains is shown in gray. Two cysteine-rich repeats inside the N-terminal domain are shown in blue. Amino acid residues above 85% identity threshold are colored according to their physicochemical properties. Styru – *Styela rustica*, Bosch – *Botryllus schlosseri*, Cioin – *Ciona intestinalis*, Ciosa – *Ciona savignyi*, Dipli – *Diplosoma listerianum*, Oikdi – *Oikopleura dioica*, Brafl – *Branchiostoma floridae*, Alvja – *Alveopora japonica*, Triad – *Trichoplax adhaerens*

The alignment of all the proteins showed high sequence similarity and a highly conserved cysteine spacing at the cysteine-rich repeats (Fig. 6). All proteins were found to contain N-terminal signal peptides, and were predicted to be secretory. Notably, rusticalin lacks 40 C-terminal residues, in contrast to its identified homologs.

**Fig. 7:**
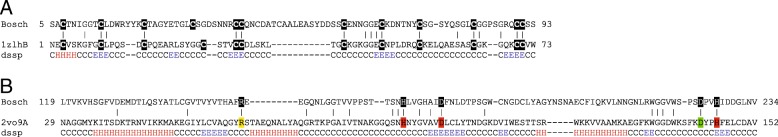
Sequence-structure alignment of N- and C-terminal domains of *Botryllus schlosseri* rusticalin sequence. **a** Alignment of the N-terminal domain with β-defensin-like fold of tick carboxypeptidase inhibitor (PDB ID 1ZLH). Conserved cysteine residues are highlighted in black. The sequence identity is 26%. **b** Alignment of C-terminal domain with Hedgehog/DD-peptidase fold of bacteriophage A500 L-alanyl-D-glutamate peptidase (PDB ID 2VO9). Amino acid residues involved in Zn^2+^ binding by 2VO9 are highlighted in red, catalytic and substrate-binding residues are highlighted in green and yellow, respectively. The sequence identity is 21%. ss_dssp – template secondary structure as determined by DSSP. The secondary structure is labeled ‘H’ for α-helix, ‘E’ for β-strand, and ‘C’ for coil

In order to characterize predicted domains of rusticalin-like proteins we performed a remote similarity search (HHpred) using a multiple sequence alignment separately for each domain as query. The multiple sequence alignment generated for individual cysteine-rich repeats (Fig. 6,) and searched with HHpred against Pfam and SCOP databases revealed similarity with β-defensin family and β-defensin-like fold (SCOP g.9.1), respectively (Fig. 7a). Additionally, the multiple sequence alignment of the N-terminal domains containing a pair of cysteine-rich repeats, searched against PDB database, showed similarity to tick carboxypeptidase inhibitor (PDB ID 1ZLH) (Fig. 7a). Remarkably, tick carboxypeptidase inhibitor is structurally related to β-defensin-like fold and is the only described protein structure comprising two β-defensin repeats. Thus, in silico analysis suggests that the N-terminal domain of rusticalin-like proteins may have a tertiary structure similar to the tick carboxypeptidase inhibitor, acting as a double-headed enzyme inhibitor [29].

In order to determine the nature of the C-terminal domain we queried its multiple sequence alignment against Pfam, SCOP, and PDB databases. The search in Pfam database showed that the C-terminal domain of rusticalin-like proteins share a significant sequence similarity with Peptidase_MD clan (Pfam ID: CL0170). Most of proteins belonging to that clan are bacterial cell-wall degradation enzymes suggesting that the C-terminal domain might originate from a bacterial genome. The search in SCOP database revealed that the C-terminal domain matched structurally with Hedgehog/DD-peptidase fold (SCOP d.65.1). Catalytic, substrate binding, and Zn-binding residues of MD peptidases were conserved (Fig. 7b), thus rusticalin-like proteins are likely to have peptidase activity. However, rusticalin itself appears to lack this activity due to the absence of 40 C-terminal residues. Finally, a high sequence similarity (Fig. 7b, 21%, E-value of 1.5E-16) of C-terminal domain with bacteriophage A500 L-alanyl-D-glutamate peptidase (PDB ID 2VO9,) indicates a possible role of bacteriophage in horizontal transfer of the C-terminal domain coding sequence from the bacterial genome.

### Evidence of a horizontal gene transfer (HGT) event

**Fig. 8:**
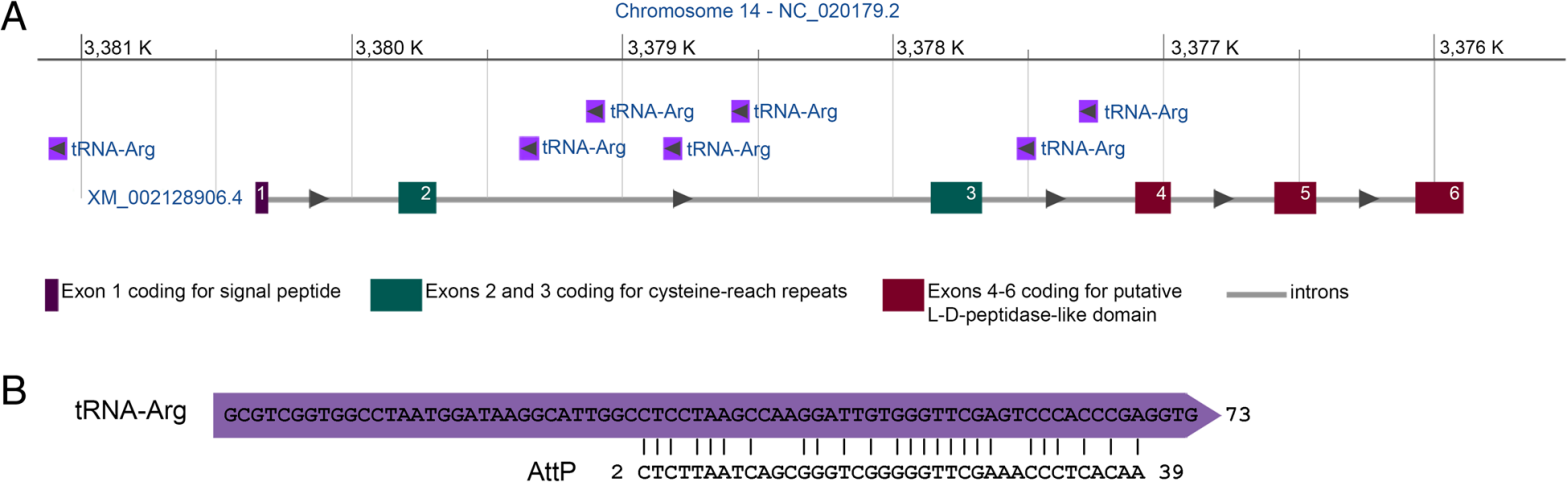
Rusticalin-like gene from *Ciona intestinalis* contains bacteriophage A500 recombination site inside the non-coding region. **a** Position and antisense orientation of seven tRNA-Arg genes inside the non-coding regions of *C. intestinalis* rusticalin-like gene (Gene ID: 100185212). The first tRNA gene is situated upstream of the protein coding sequence, four of tRNA genes are inside the second intron and two are inside the third intron neighboring the C-terminal domain coding region. **b** Alignment of bacteriophage A500 recombination site (AttP) with *Ciona intestinalis* tRNA gene (Gene ID: 108950122) situated inside the third intron. The sequence identity and score are, respectively, 65.8% and 73 (calculated by EMBOSS Matcher)

**Fig. 9:**
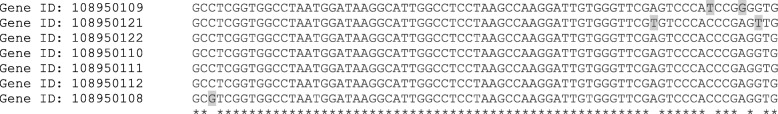
Alignment of seven *Ciona intestinalis* tRNA genes neighboring rusticalin-like gene coding sequence. Asterisks indicate conservative positions. Three of the tRNA genes differ from the other tRNA genes at four nucleotide positions (shaded grey). The sequence identity is 95–100%

**Fig. 10:**
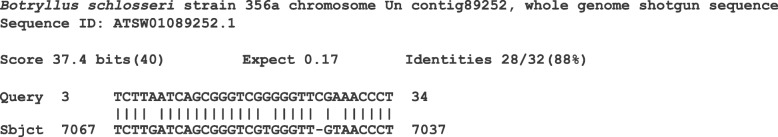
Result of nucleotide BLAST of bacteriophage A500 recombination site (AttP) against *Tunicata* genome sequences. Alignment of AttP site with *Botryllus schlosseri* genome sequence - contig89252. The sequence identity is 88%, E-value: 0.17

Bacteriophage A500 site-specific recombination involves the 3′ region of the bacterial tRNA gene [30]. Thus all genomes containing rusticalin-like proteins were searched for tRNA genes neighboring rusticalin-like genes. The rusticalin-like gene of *C. intestinalis* (Gene ID: 100185212) contains seven tRNA genes at antisense orientation situated upstream of the gene and inside the second and third introns from 5′-end (Fig. 8a). Multiple sequence alignment shows that seven tRNA genes of *C. intestinalis* are highly similar, with the sequence identity from 95 to 100% (Fig. 9). The third intron containing tRNA genes is adjacent to the C-terminal domain. Alignment of tRNA gene (Gene ID: 108950108) lying inside the third intron with bacteriophage A500 recombination site (AttP) showed the presence of a similar sequence (Fig. 8b). Thus in *C. intestinalis* rusticalin-like protein L-alanyl-D-glutamate peptidase domain is adjacent to the intron containing a region resembling the bacteriophage recombination site, confirming the domain’s horizontal transfer by means of a viral genome. We also conducted a nucleotide BLAST of the bacteriophage AttP site against all *Tunicata* genomic sequences, which gave a hit with *B. schlosseri* contig89252 (Fig. 10). We can conclude that a sequence similar to bacteriophage A500 AttP is present in *Tunicata* genomes. Nucleotide BLAST against *T. adhaerensis* genome gave no positive results.

**Fig. 11:**
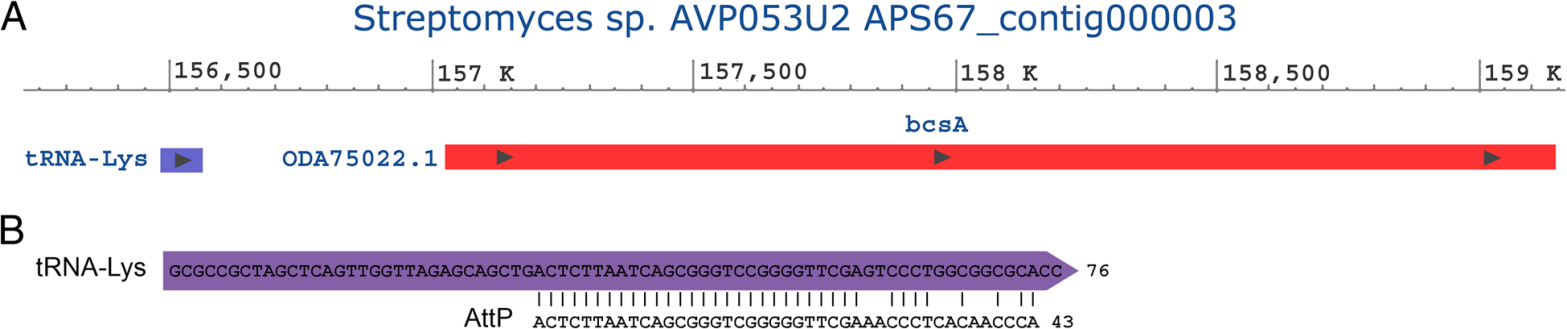
Donor of ascidian cellulose synthase *Streptomyces sp.* contains bacteriophage recombination site adjacent to cellulose synthase gene. **a** *Streptomyces sp.* cellulose synthase catalytic subunit gene (bcsA) is adjacent to tRNA-lys gene. **b** Alignment of bacteriophage A500 recombination site (AttP) with the tRNA-Lys gene (APS67_000733). The sequence identity and score are, respectively, 91.2% and 143 (calculated by EMBOSS Matcher)

We analyzed the genome of *Streptomices* sp., the prokaryote donor of ascidian cellulose synthase gene [5]. Cellulose synthase catalytic subunit gene (bcsA) was found to be adjacent to tRNA-Lys gene in this genome (Fig. 11a). Pairwise alignment of the tRNA gene with bacteriophage A500 AttP showed the presence of a highly similar sequence (Fig. 11b). This result suggests the involvement of tRNA gene in HGT of cellulose synthase into the tunicates genome.

**Fig. 12:**
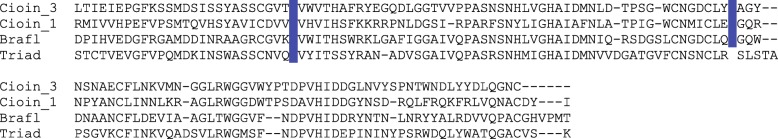
Alignment of C-terminal domains of rusticalin-like proteins with designated positions of introns. Blue vertical bars represent the positions of introns in the corresponding DNA sequence of: Cioin_3 – *Ciona intestinalis* XP_002122335.1; Cioin_1 – *Ciona intestinalis* XP_002128942.1; Brafl – *Branchiostoma floridae* XP_002588042.1; Triad – *Trichoplax adhaerens* XP_002117795.1

Taking into account that bacteriophage A500 genes do not contain introns [30] we used the presence of introns and their positions to predict the number of independent HGT events. Information about the positions of introns was available for *C. intestinalis*, *B. floridae,* and *T. adhaerens* rusticalin-like genes. The positions were mapped on the corresponding protein sequences. Two introns were found to be located inside the C-terminal domain coding region (Fig. 12), and their positions were strictly conserved in the sequences analyzed. This fact suggests that the C-terminal domain was formed as a result of a single gene transfer event of L-alanyl-D-glutamate peptidase. Synonymous distances counted between bacteriophage A500 enzyme and C-terminal domain of those four proteins indicated that the shortest distance of 34 substitutions is in bacteriophage A500 and *C. intestinalis* (Cioin_1) comparison. Based on this data we speculate that the first acceptor of a foreign gene belonged to the Tunicata lineage.

## Discussion

### Specific expression of rusticalin in hyalinocytes

As previously shown, percoll gradients are suitable for isolation of cell populations in marine invertebrates. They have been successfully used for identification of cell-type-specific proteins through antibody (AB) production [23, 31] or by MALDI MS\MS analysis with subsequent RACE PCR [32, 33]. About 40% of the blood cells in the ascidian *Styela rustica* are represented by hyalinocytes [22]. Hyalinocytes or their equivalents in other ascidian species perform functions such as phagocytosis [22, 27], cytokine synthesis [34], and protease release upon LPS induction [28]. In order to isolate the rusticalin protein of hyalinocytes and describe its gene, we conducted MALDI and RACE. DNA-RNA FISH of the newly identified gene confirmed its specific expression in hyalinocytes (Fig. 4). The rusticalin gene with the deduced amino acid sequence was compared to other genome and transcriptome sequences from many species using both BLAST search and methods specialized for remote similarity search – Hhblits and HHpred. This approach allowed us to characterize a new protein, rusticalin, and predict properties for rusticalin as well as for group of homologous rusticalin-like Proteins. Rusticalin-like proteins are present in basal chordates and, also in primitive multicellular animals: coral *A. japonica* and placozoan *T. adhaerens*.

### Putative function of rusticalin-like proteins

Prediction of protein disorder and solvent accessibility for rusticalin showed the existence of two distinct structural domains (Fig. 3). N-terminal domain contained two cysteine-rich repeats. Querying of sequence and predicted structure of cysteine-rich repeats in protein databases showed that they resembled β-defensins, antimicrobial proteins responsible for the lysis of pathogens [35–37] by disrupting their membranes [38]. On the other hand, the C-terminal domain of rusticalin-like proteins was identified as a Peptidase_MD clan member and, more specifically, as being close to L-alanyl-D-glutamate peptidase. Catalytic, substrate binding, and Zn-binding sites of the enzyme [39] were conserved in all rusticalin-like proteins suggesting that they may have peptidase activity. Other members of MD peptidases are bacterial cell-wall digesting enzymes [39–42]. Though the precise function of rusticalin-like proteins cannot be identified yet, we may venture a guess that the N-terminal domain perforates bacterial cell walls while the C-terminal domain digests them. Accordingly, all rusticalin-like proteins are predicted by TargetP to be secretory. The fact that rusticalin is specific to hyalinocytes does not contradict its putative immune function since at least hyaline amoebocytes are also known to be capable of phagocytosis [27]. Another protein previously characterized as Zn-dependent metallo-protease from the ascidian *Halocynthia roretzi* hemocytes is activated by lipopolysaccharide (LPS) [28, 43, 44] and hence might also be involved in immune reactions.

At the same time, a pair of the cysteine-rich repeats analyzed separately showed a significant similarity with carboxypeptidase inhibitor of the tick *Rhipicephalus bursa*. This protein is also related to β-defensin-like fold [29] but its function is to inhibit carboxypeptidase-A/B of mammalian blood [45]. Based on this finding, we propose an alternative scenario for the interaction of the N- and C-terminal domains, where the N-terminal domain exerts no bactericidal function but acts as a regulatory subunit. This mode of interaction has been described for carboxypeptidases A/B (M14) [46], for zinc-dependent matrix metalloproteases (MMPs) [47], and also for LytM [48], which is related to MD peptidases [49]. Thus, the ancestral state of the N-terminal domain’s function might have been the perforation of the bacterial membrane. Whatever the case, putative functions of the newly described protein should be verified experimentally by production of recombinant protein. Rusticalin of *S. rustica* is 40 amino acids shorter and lacks a part of the active site. This means that it cannot perform an enzymatic function but might still be involved in the signaling pathways of the immune reaction [50, 51], similarly to the Hedgehog signaling molecule, another member of peptidase MD family [52].

### Possible horizontal gene transfer (HGT)

Cellulose synthase of the ascidian *C. intestinalis* provides one of the clearest examples of HGT [5]. In the present study we described another ascidian protein, rusticalin, whose C-terminal domain probably originated by means of HGT from a bacterial cell-wall digesting enzyme. Moreover similarity with bacteriophage A500 L-alanyl-D-glutamate peptidase suggests a possible involvement of a bacteriophage as a vector. This hypothesis is supported by the fact that the C-terminal domain belongs to bacterial MD peptidases (Pfam ID CL0170) and at the same time shows significant sequence similarity with bacteriophage protein (E-value 1.5e-16). It is further confirmed by an analysis of noncoding regions of *C. intestinalis* rusticalin-like gene, which contained a sequence similar to the bacteriophage A500 recombination site [30]. While many cases of HGT are described based on sequence similarity alone [15, 53–59], in the case of rusticalin we also demonstrated strong evidence of the mechanism of transfer by identifying the recombination site.

Rusticalin-like proteins are also present in a primitive multicellular animal *Trichoplax adhaerens* [60, 61] and the coral *Alveopora japonica.* However, no remains of bacteriophage A500 recombination sites were found in the *T. adhaerens* or *A. japonica* nucleotide sequences. The signatures of the bacteriophage gene transfer might have been erased from the *T. adhaerens* genome as a result of intron shortening [61] (Table 2) but preserved in the *C. intestinalis* genome, possibly, due to the possession of functioning tRNA genes inside the introns (Fig. 7). We also found that *Streptomices sp.*, the prokaryote donor of the ascidian cellulose synthase gene [5], contained tRNA-Lys gene and a sequence similar to the bacteriophage recombination site (AttP) adjacent to the cellulose synthase catalytic subunit gene (bcsA). This fact supports the hypothesis that viral recombination with tRNA genes was involved in HGT events and suggests a common mechanism for at least these two cases of HGT.

**Table 2:** Intron length in rusticalin-like genes

intron# from 5′		Intron length bp				
		1	2	3	4	5
Species	Cioin_1	494	1828	573	388	369
	Cioin_3	317	168	225	68	190
	Brafl	–	1139	718	460	646
	Triad	200	164	287	164	–

*Cioin Ciona intestinalis*, *Brafl Branchiostoma floridae*, *Triad Trichoplax adhaerens*. The intron length is underlined for introns containing tRNA genes

Since *T. adhaerens, A. japonica,* and Chordata are distant animal relatives [62], it can’t be ruled out that HGT events for C-terminal domains of their rusticalin-like proteins were independent. Still, the position of the fourth intron inside the C-terminal domain coding region is identical for the placozoan *T. adhaerens*, the ascidian *C. intestinalis,* and the cephalochordate *B. floridae*. Given that the genome of the bacteriophage A500 contains no introns [30], they must have been introduced right after the gene transfer to the eukaryote genome [63]. It seems improbable that the identical intron positions are the result of an independent intron gain. Thus, we assume that the fourth intron appeared as a result of a single event of intron insertion into the C-terminal domain coding region. This means, in turn, that the C-terminal domain of rusticalin and rusticalin-like proteins emerged as a result of a single HGT event of L-alanyl-D-glutamate peptidase, inserted by the bacteriophage into the eukaryote genome. We performed a synonymous distance analysis between the bacteriophage A500 enzyme and the C-terminal domains of four rusticalin-like proteins that possess the identical intron positions. The *C. intestinalis* gene (Cioin_1) appeared to have shortest synonymous distance to the bacteriophage enzyme. The same gene contains a tRNA and a sequence similar to the AttP site inside its introns. Thus, this supports the hypothesis that the first HGT event mediated by a bacteriophage happened in the Tunicata lineage.

## Conclusion

We described a new protein, rusticalin, from the hyalinocytes of the ascidian *Styela rustica* and predicted its features based on the sequence analysis. Discernible homologues of rusticalin were found only in basal chordates, coral, and placozoans. Sequence similarity and the presence of a putative bacteriophage recombination site support the hypothesis of transfer of the C-terminal domain from a bacteriophage genome. A similar mechanism involving bacteriophage as a vector can be proposed for the cellulose synthase catalytic subunit gene.

## Methods

### Animals

Ascidians *Styela rustica* Linnaeus (1767) were collected off Fettakh Island near the Biological Station of the Zoological Institute of the Russian Academy of Sciences at Cape Kartesh (Kandalaksha Bay, the White Sea) in June–August of 2013–2017. The ascidians were kept in cages at a depth of 3–4 m throughout the experimental period.

### Collection of hemocytes

All manipulations with ascidians were carried out in a temperature-controlled room at 10 °C. Before bleeding, the animal was washed with sea water and dried with absorbent paper. Then the sampling area was sterilized with 70% ethanol and the ascidian body wall was cut with a razor blade to the muscular layer without injuring the internal organs. Hemolymph was collected from the cut with a micropipette and transferred into a tube containing an anticoagulant solution (AS) (0.3 M NaCl, 20 mM KCl, 15 mM EDTA, 10 mM HEPES pH 7.6) [23].

### Discontinuous percoll gradient for hemocytes fractionation of hemocytes

Percoll solution (Sigma) was mixed with appropriate volumes of AS to obtain final concentrations of 60, 45, and 35%. Three milliliters of each mixture was overlaid sequentially into a glass centrifuge tube. The blood sample was made by pooling blood from four animals and mixing it with AS (1:1). Three milliliters of the blood sample was layered onto the percoll gradient and the tube was centrifuged in a swing rotor at 800 g for 30 min. Cells from the density boundary were collected by gentle aspiration and washed thrice in AS. The cell composition of fractions was determined by phase-contrast microscopy. The protein composition of the fractions was analyzed by SDS-PAGE.

### SDS-page

Protein samples for SDS-PAGE were prepared out of whole blood cells or cell fractions after separation in percoll gradient. Cells were centrifuged at 800 g for 10 min, resuspended in 7 mM EDTA, 1 mM PMSF, 10% β-mercaptoetanol, and frozen (− 20 °C). After thawing the suspension was mixed with 2x loading buffer (0.3 Tris-HCl pH 6.8; 20% glycerol; 4% SDS; 5% β-mercaptoetanol) and boiled for 5 min. SDS-PAGE was performed on 15% gels with Mini-Protean II electrophoretic cell (Bio-Rad). Unstained Protein MW marker (Thermo Scientific) was used as a size standard. To visualize proteins, the gel slabs were stained with Coomassie BB R-250 (Biolot, Russia).

### Protein sequencing and tandem mass spectrometry

After SDS-PAGE of whole blood cells proteins were transferred to PVDF membrane and stained with Ponceau S (Fig. 1, line 4). A protein band of apparent molecular mass 23 kDa was excised and subjected to Edman degradation (Alta Bioscience, interior code of sample: S6269, Birmingham, UK). This method provided no accurate amino acid sequence. Therefore, an equal protein band was excised from polyacrylamide gel and subjected to digestion with Proteomics Grade Trypsin (Sigma). Tryptic fragments were further extracted from the gel matrix and analyzed by MALDI MS/MS at PostGenome analysis center (http://xn--h1aaoah.xn--p1ai/services-and-rates/mass-spectrometry.html, Moscow). The resulting partial amino acid sequence was used to create nested degenerate oligonucleotide primers designed with iCODEHOP [64].

### Cloning and sequencing of rusticalin cDNA

Total RNA was extracted from blood cells of *S. rustica* using TRI Reagent (Sigma) and reverse-transcribed with MINT cDNA synthesis kit (Evrogen) according to the manufacturer’s instructions. MINT RACE cDNA Amplification Set (Evrogen) was used for 3′ and 5’ RACE. For 3’RACE, nested degenerate oligonucleotide primers were designed using the iCODEHOP algorithm [64] on the basis of the determined amino acid sequence (Table 3; #1, 2). Primers for 5′RACE (Table 3; #3, 4) were based on the DNA sequence obtained in 3’RACE. Both 3′ and 5’PCR products were cloned in pAL2-T vector, using Quick-TA kit (Evrogen, Russia), and Sanger sequenced in Evrogen.

**Table 3:** Oligonucleotide primers used in the study

	Primer	Sequence 5′-3′
1	Sr_P26_3’_F1	ggnaa**ywsntayathmng**
2	Sr_P26_3’_F2	**tasttayattcgt**tgt
3	Sr_P26_5’_R1	cattgtgccaagttc**ccgag**
4	Sr_P26_5’_R2	**ccgag**caatggttgctgttta

h = a,c,t; y = c,t; s = c,g; n = a,g,c,t; m = a,c; w = a,t. Bold letters with underscore – overlaying parts of nested primers

### DNA-RNA fish

Two synthetic 26–27-mer 5′-end biotin-labeled DNA probes were used for DNA-RNA FISH. Probe 1 (/Biotin/CAGTTGTTGCTCATAACCGGCGATGC-3′) was complementary to 113–138 nucleotide region corresponding to N-terminal domain of rusticalin, while Probe 2 (/Biotin/GGCGACTCGAATTACCTTGCCCTGATA-3′) was complementary to 400–426 nucleotide region corresponding to the C-terminal domain of rusticalin. Hybridization without probe served as negative control.

Ascidian blood was collected as described above. Blood drops were transferred from the cut in the body wall directly onto a glass slide (Superfrost Plus, Menzel) and left for 20 min at 10 °C for cell attachment. The cells were fixed with 4% PFA in AS for 10 min at 10 °C and washed successively in AS, distilled water, and methanol. The slides were dried and stored frozen (− 20 °C) until use. For morphological control several slides with spread cells were resolved and stained with hematoxylin and eosin, dehydrated, and embedded in Dammar resin. Images were taken on Leica DM6000 with DIC (Nomarsky optics).

Before FISH the excessive PFA was washed off with PBT (1 × PBS, 0.1% Tween 20). Cells were pretreated with 2 μg·ml^− 1^ proteinase K (Thermo Scientific), 0.1% SDS in PBS for 2 min. The proteinase K was then inactivated by incubation with 200 μM PMSF. Cells were postfixed in 4% PFA and washed again with 200 μM PMSF. Excessive PFA was washed off with PBT. Endogenous biotin was blocked as described by Miller and Kubier [65]. The cells were then washed thrice for 10 min with PBS and postfixed in 4% PFA. Excessive PFA was washed with PBT.

To perform DNA-RNA FISH the cells were rinsed in 4 × SSC and prehybridized in hybridization buffer (1% dextran sulfate, 50% formamide, 1 mg·ml^− 1^ salmon sperm DNA in 4 × SSC) for 15 min at 36 °C. Hybridization was performed with 0.5 μM of probe in hybridization buffer for 17 h at 36 °C. After hybridization the samples were washed in 50% formamide, 4 × SSC at 36 °C and then in 0.2 × SSC, 0.1% Tween 20 at 45 °C. After blocking in 1× In Situ Hybridization Blocking solution (Vector laboratories) in PBT at 37 °C for 60 min, the probe was detected using strepavidin-Alexa594 (1:500, Life technologies) at 37 °C for 120 min. The samples were washed thrice at 37 °C in PBT, counterstained with 3 μg/ml DAPI and mounted in 80% glycerol in 1 × PBS. Fluorescent images were taken with the use of confocal laser microscope LEICA TCS SP5 MP.

### Sequence analysis and database searches

The workflow of sequence analysis and database searches is shown in Fig. 5. The average molecular mass and isoelectric point of rusticalins were calculated with ProtParam [66] on the ExPASy server (https://www.expasy.org/). Signal peptides were predicted with Phobius at EMBL-EBI [67] and SignalP [68]. Subcellular location was predicted with SCL-Epred [69] and TargetP [70]. Globular domains were predicted with Scooby-domain. Internal repeats were identified with REPRO [71] and RADAR [72] algorithms. Relative solvent accessibility was predicted with PaleAle [73]. Disordered regions were predicted with Disopred3 [74] and SPOT-disorder [75], and protein backbone dynamics was predicted with DynaMine [76]. All secondary structure predictions were made after removal of the signal peptide.

The initial tBLASTn searches were performed against transcriptome database (EST) available at NCBI server. HHblits [77] was used to search in UniProtKB and the NCBI non-redundant protein databases. Obtained hits showing both conservation of cysteine residues and more than 90% sequence coverage were trimmed to remove putative signal peptide and aligned using MSAProbs [78]. The aligned sequences were filtered to 90% identity and subjected to remote similarity searches using HHpred [79] in PDB, SCOP, and Pfam 30.0 protein databases. Multiple sequence alignment was visualized with CHROMA software [80].

### Genomic sequences and gene structure

tRNA genes positions in genomic sequences were retrieved from whole-genome shotgun sequences of *Ciona intestinalis* (GCA_000224145.2) and *Streptomices sp*. AVP053U2 isolated from *Styela clava* (LMTQ02000003.1) [81]. Sequences of seven *C. intestinalis* tRNA genes (Gene ID: 108950112, 108,950,111, 108,950,110, 108,950,109, 108,950,108, 108,950,122, 108,950,121) were obtained from NC_020179.2 genome region (Chromosome 14). Sequences of *Streptomices sp*. tRNA gene (APS67_000733) were obtained from region 156,485–156,560 of contig000003. tRNA genes were aligned using the Clustal Omega multiple sequence alignment program [82]. Pairwise alignment of bacteriophage recombination site sequence and tRNA genes was made in EMBOSS Matcher [83]. Database searches restricted to *Tunicata* were performed using BLASTn against GenBank nucleotide collections: nr/nt database, expressed sequence tags (EST), and whole-genome shotgun contigs (WGS).

Information about gene structure was available in GenBank for four rusticalin-like sequences: two *Ciona intestinalis* genes GeneID:100181995, XM_002122299.4, XP_002122335.1 and GeneID:100185212, XM_002128906.4, XP_002128942.1; *Branchiostoma floridae* gene GeneID:7231622, XM_002587996.1, XP_002588042.1 and *Trichoplax adhaerens* gene GeneID:6759007, XM_002117759.1, XP_002117795.1. Intron positions were mapped on the corresponding amino acid sequences preserving alignment.

The same gene sequences with addition of bacteriophage A500 gene (GeneID:5601386) were used to calculate synonymous distances with SNAP v2.1.1 [84]. Distances were calculated based on codon-alignment preserving alignment of amino acid sequences.

## Abbreviations

AB: Antibodies, LPS – lipopolysaccharide
AS: Anticoagulant solution
DAPI: 4′-6-Diamidino-2-phenylindole
FISH: Fluorescent in situ hybridization
HGT: Horizontal gene transfer
MALDI MS/MS: Matrix-assisted laser desorption/ionization tandem mass spectrometry
MMP: Matrix metalloprotease
PBT: 1×PBS, 0.1% Tween 20
RACE: Rapid Amplification of cDNA Ends
